# Non-invasive assessment of hepatic fibrosis in pediatric survivors of acute lymphoblastic leukemia: a cross-sectional study

**DOI:** 10.1007/s00431-026-07205-w

**Published:** 2026-07-24

**Authors:** Laila M. Sherief, Mohamed A. Almalky, Sameh S. Bayoumi, Hosam E. Salah, Basma K. Soliman, Naglaa M. Kamal, Sherif M. Talaat, Hanan S. Sherbiny, Asmaa D. Alsufyani, Faisal S. Alosaimi, Enas A. A. Abdallah

**Affiliations:** 1 Department of Pediatrics, Faculty of Medicine, Zagazig University, Zagazig, Egypt; 2 Department of Radio-diagnosis, Faculty of Medicine, Zagazig University, Zagazig, Egypt; 3 Department of Clinical Pathology, Faculty of Medicine, Zagazig University, Zagazig, Egypt; 4 Department of Pediatrics and Pediatric Hepatology, KasrAlainy Faculty of Medicine, Cairo University, Cairo, Egypt; 5 Department of Pediatrics, Alahrar Teaching Hospital, Zagazig, Egypt; 6 Department of Child Health, College of Medicine, University of Bisha, Bisha, Saudi Arabia; 7 Department of Pediatrics, Taif Children’s Hospital, Taif, Saudi Arabia; 8 Department of Pediatrics, Alhada Armed Forces Hospital, Taif, Saudi Arabia

**Keywords:** Liver fibrosis, Acute lymphoblastic leukemia, Transient elastography, Enhanced liver fibrosis, Hepatitis C virus, Pediatric survivors

## Abstract

Survival rates for pediatric acute lymphoblastic leukemia (ALL) have markedly improved; however, survivors remain at risk of long-term hepatic complications, including progressive fibrosis related to chemotherapy and prior viral infections. The objective of this study is to evaluate hepatic fibrosis in pediatric ALL survivors using a sequential non-invasive algorithm combining transient elastography (TE) and enhanced liver fibrosis (ELF) score. This comparative cross-sectional study included 159 pediatric ALL survivors who had completed therapy at least 2 years earlier and 99 age- and sex-matched healthy controls from two Egyptian oncology centers. Liver fibrosis was assessed using TE and serum biomarkers for calculation of the ELF score. The mean age at diagnosis was 6.9 ± 2.7 years, and at assessment, 11.1 ± 2.7 years. Hepatitis C virus (HCV) prevalence declined significantly from 75% during treatment to 5% at enrollment following antiviral therapy (*p* < 0.001). Fibrosis was detected in 75% of survivors by TE, predominantly moderate (F2) fibrosis. Combined TE/ELF assessment identified advanced fibrosis (≥ F3) in 6% of patients, while most survivors had mild-to-moderate fibrosis. Mean ELF score was significantly higher in survivors than controls (9.3 ± 1.2 vs. 4.5 ± 1.03, *p* < 0.0001). Agreement between TE and ELF was fair (Cohen’s *κ* = 0.34–0.39). HCV-positive survivors had significantly higher ELF scores than HCV-negative peers.

*Conclusion*: Persistent hepatic fibrosis in pediatric ALL survivors was observed predominantly among patients with current or previous HCV exposure. A combined TE/ELF approach represents an effective non-invasive strategy for early detection and monitoring.

**What is Known**

• *Hepatic complications are expected among ALL survivors due to significant exposure to medications and viral infections*.

**What is New**

•*Using sequential non-invasive algorithm combining transient elastography (TE) and enhanced liver fibrosis(ELF) revealed variable stages of hepatic fibrosis among 75% of ALL survivors particularly current or previous HCV positive patients. Moderate fibrosis was the most prevalent stage. HCV was successfully eradicated after treatment with DAAs medications in almost all treated pediatric patients.*

## Introduction

Acute lymphoblastic leukemia (ALL) accounts for approximately 30% of all pediatric malignancies and up to 70% of pediatric leukemias in Egypt [1]. Survival rates have improved dramatically over recent decades, rising from nearly zero in the 1950 s to over 95% today due to advances in therapeutic strategies [2]. Despite these improvements, survivors remain at increased risk of long-term complications, including hepatic injury, largely attributable to the non-specific nature of curative therapies [3] and potential exposure to hepatotropic viruses during treatment [4].

Progression to liver fibrosis and cirrhosis is a major concern in this population, particularly given the immunosuppressive effects of cancer therapy. Early detection and accurate assessment of hepatic fibrosis are therefore essential to guide follow-up and prevent serious complications such as cirrhosis, hepatocellular carcinoma, liver transplantation, and liver-related mortality [5].

Liver biopsy has historically been considered the reference standard for assessing hepatic fibrosis; however, its limitations—including invasiveness, sampling variability [6], interobserver variability [7], and inability to reflect the dynamic nature of fibrosis [8]—have reduced its acceptability, particularly for longitudinal monitoring [9]. Although still regarded as the “best available” method [10], increasing evidence supports the use of reliable non-invasive alternatives [11].

Serum biomarkers reflecting extracellular matrix turnover provide a non-invasive approach to fibrosis assessment [12]. The enhanced liver fibrosis (ELF) score combines hyaluronic acid, amino-terminal propeptide of type III collagen, and tissue inhibitor of metalloproteinase-1 and has been validated across a range of chronic liver diseases [11, 13–15]. However, its lack of liver specificity and limited accuracy in intermediate fibrosis stages restrict its use as a standalone diagnostic tool [16].

Transient elastography (TE) is a widely used, non-invasive imaging modality that measures liver stiffness and correlates well with histological fibrosis staging [17]. It provides rapid and reproducible results and is particularly useful in the assessment of chronic liver diseases, including hepatitis C infection [17–20]. However, TE measurements may be affected by factors such as acute inflammation, congestion, obesity, and technical limitations, especially in pediatric patients [21, 22].

To improve diagnostic accuracy and reduce reliance on liver biopsy, recent research has focused on combining different non-invasive modalities. The sequential use of TE and ELF has demonstrated high diagnostic performance in both the detection and monitoring of liver fibrosis, including in patients undergoing antiviral therapy for HCV [11, 15, 21–24].

In this context, the present study aimed to evaluate hepatic fibrosis among pediatric survivors of ALL using a combined non-invasive approach based on TE and ELF score, and to assess the impact of HCV exposure on fibrosis severity in this population.

## Subjects and methods

### Study design and setting

This comparative cross-sectional study was conducted at the Pediatric Hematology Oncology unit, Pediatric Department,Zagazig University Children’s Hospital, Zagazig, Egypt, andoncology Department, Benha Specialized Children’s Hospital, Egypt. The study was carried out in accordance with the Declaration of Helsinki.

### Participants

The study included 159 pediatric survivors of acute lymphoblastic leukemia (ALL) who had completed treatment according to the St. Jude Total XV protocol [25] at least 2 years prior to enrollment. Participants were recruited over a 2-year period and constituted the case group.

A total control group of 99 apparently healthy children, matched for age and sex, was included after exclusion of one participant with previously unrecognized HCV positivity and abnormal fibrosis findings who was excluded from the final analysis.

Inclusion criteria comprised ALL survivors of both sexes who had completed therapy at least 2 years earlier. Exclusion criteria included patients who were newly diagnosed, still receiving treatment, had incomplete medical records, or had pre-existing chronic liver disease prior to ALL diagnosis.

### Ethical approval

The study was approved by the Institutional Review Board (IRB) and the Research Ethics Committee of the Faculty of Medicine, Zagazig University, Egypt. Written informed consent was obtained from the parents or legal guardians of all participants.

### Clinical assessment

All participants underwent detailed history taking and thorough clinical examination. Anthropometric measurements, including weight, height, and body mass index (BMI), were recorded and plotted according to Centres for Disease Control and Prevention (CDC) growth charts [26].

Medical records were reviewed to collect data on age at diagnosis, treatment protocol, clinical course, complications, duration since treatment completion, and hepatitis B and C status. For HCV-positive patients, details of antiviral therapy and post-treatment status were documented.

### Laboratory investigations

Blood samples were collected under aseptic conditions for routine laboratory investigations, including complete blood count (CBC), liver and kidney function tests, and HCV quantification.

CBC was performed using a Sysmex KX-21 analyzer with peripheral blood smear examination [27]. Liver and kidney function tests were conducted using the Dimension e.S. auto-analyzer [28]. HCV RNA quantification was performed using real-time polymerase chain reaction (PCR) (COBAS Amplicor 2.0, Roche Diagnostics, USA), with a lower detection limit of 10 IU/mL.

### Transient elastography (TE)

All participants underwent transient elastography (Fibroscan) performed by a single experienced radiologist using a standardized protocol.

Liver stiffness measurement (LSM) was obtained using a 5-MHz probe. The procedure was performed with the patient in the supine position, with the right arm in maximal abduction. Measurements were taken from the right lobe of the liver through the intercostal space.

At least ten valid measurements were obtained for each participant, with a success rate of ≥ 60% and an interquartile range (IQR) of ≤ 30%. The median value was recorded in kilopascals (kPa).

Fibrosis staging was based on the METAVIR scoring system, ranging from F0 (≤ 6 kPa) to F4 (≥ 9 kPa), with significant fibrosis defined as ≥ F2 [29].

### Enhanced liver fibrosis (ELF) score

Serum samples were collected, centrifuged, and stored at − 20 °C for analysis of fibrosis biomarkers.

The following biomarkers were measured using enzyme-linked immunosorbent assay (ELISA):

- Hyaluronic acid (HA)
- Amino-terminal propeptide of type III collagen (PIIINP)
- Tissue inhibitor of metalloproteinase-1 (TIMP-1)

The ELF score was calculated using the validated formula:

ELF = 2.278 + 0.851 ln(HA) + 0.751 ln(PIIINP) + 0.394 ln(TIMP-1) [30].

Cutoff values of 7.7 and 9.8 were used to classify fibrosis into mild, moderate, and severe stages, while pediatric advanced liver fibrosis (> F3) ELF threshold of ≥ 10.51 is previously validated [24] Click or tap here to enter text.

### Combined TE/ELF assessment

A sequential algorithm based on previous studies was applied to improve diagnostic accuracy [17].

Low values of either TE (≤ 6 kPa) or ELF (≤ 7.7) were used to exclude advanced fibrosis (> F3). High values of both TE (≥ 9 kPa) and ELF (≥ 10.5) confirmed advanced fibrosis.

Cases with intermediate results were classified according to the complementary test. Fibrosis stages were categorized as follows:

- Mild (F0–F1)
- Moderate (F2)
- Severe (F3)
- Advance (> F3/F4)

This combined approach has demonstrated high diagnostic accuracy compared with liver biopsy [24].

### HCV terminology

HCV infection was defined by detectable HCV RNA using a PCR assay, while sustained virologic response was defined as undetectable HCV RNA 12 weeks after completion of antiviral therapy.

### HCV management

All HCV-positive patients received direct-acting antiviral (DAA) therapy according to national guidelines. A combination of ledipasvir and sofosbuvir was administered once daily for 12 weeks in children older than 3 years, as part of the national initiative toward HCV elimination in Egypt [2].

### Statistical analysis

Data were analyzed using SPSS version 16 (SPSS Inc., Chicago, IL, USA). Quantitative data were expressed as mean ± standard deviation (SD), median, and range, while categorical variables were presented as frequencies and percentages.

Normality was assessed using the Kolmogorov–Smirnov test. Comparisons between groups were performed using Student’s *t*-test for normally distributed variables and the Mann–Whitney *U* test for non-parametric data. Categorical variables were analyzed using the chi-square test or Fisher’s exact test.

Correlation between TE and ELF was assessed using Spearman’s correlation coefficient and Cohen’s kappa. A *p*-value ≤ 0.05 was considered statistically significant.

## Results

### Baseline characteristics

The study included 159 pediatric survivors of acute lymphoblastic leukemia (ALL) and 99 age- and sex-matched healthy controls. Among survivors, 81.2% had B-cell ALL, and 52.5% were male. The mean age at diagnosis was 6.9 ± 2.7 years, and the mean age at enrollment was 11.1 ± 2.7 years, with a mean follow-up duration of 4.2 ± 1.9 years.

No significant differences were observed between cases and controls regarding age, sex, or body mass index. However, hepatomegaly and splenomegaly were significantly more frequent among ALL survivors (Table 1).

**Table 1:** Biodemographic and physical findings among study participants

Variable	ALL survivors (*n* = 159)	Controls (*n* = 99)	Test of significance	*P*
Age at ALL diagnosis (ys), mean ± SD	6.9 ± 2.7	-	-	-
Age at enrollment (ys), mean ± SD	11.1 ± 2.7	10.9 ± 2.8	*t* = 0.16	0.87
Gender				
Male, *N* (%)	83 (52)	49 (49.5)	*χ*^2^ = 0.067	0.79
Female, *N* (%)	76(48)	50 (50.5)		
BMI (kg/m^2^), mean ± SD	19.7 ± 5.3	19.1 ± 5.25	*t* = 1.84	0.066
Hepatomegaly, *N* (%)	22 (14)	5(5)	*χ*^2^ = 4.23	0.039
Splenomegaly, *N* (%)	8 (5)	0 (0)	*χ*^2^ = 13.2	< 0.0001

### Laboratory findings and HCV/HBV status

There were no significant differences between groups in most hematological and biochemical parameters. However, alanine aminotransferase (ALT) and aspartate aminotransferase (AST) levels were significantly higher in ALL survivors compared with controls (Table 2).

**Table 2:** Laboratory investigations, HCV status, and fibrosis staging of the populations studied

Variable	ALL survivors (*n* = 159)	Controls (*n* = 99)	Test of significance	*P*
Hb (gm/dl), mean ± SD	11.9 ± 0.90	12.0 ± 0.62	0.47	0.64
WBCs (× 10^3^), mean ± SD	7.26 ± 1.94	7.33 ± 1.69	0.19	0.85
PLTs (× 10^3^), mean ± SD	273.0 ± 60.53	283.6 ± 49.65	0.95	0.34
ALT, mean ± SD	40.2 ± 34.8	21.7 ±.8.2	2.95	0.003
AST, mean ± SD	38.6 ± 24.4	24.5 ± 12.7	3.07	0.002
Albumin, mean ± SD	4.34 ± 0.32	4.38 ± 0.26	− 0.273	0.779
Total.Bil, mean ± SD	0.59 ± 0.15	0.61 ± 0.19	− 0.291	0.773
Direct. Bilirubin, mean ± SD	0.16 ± 0.06	0.17 ± 0.055	− 0.320	0.750
Creatinine, mean ± SD	0.59 ± 0.15	0.53 ± 0.14	2.36	0.018
HCV positive at the end of ALL treatment, *N* (%)	119 (75)	_	__	_
HCV positive at enrollment, *N* (%)	8 (5)	-	–	-

A high prevalence of hepatitis C virus (HCV) infection was documented by the end of ALL treatment (75%). At enrollment, HCV positivity declined significantly to 5% following treatment with direct-acting antivirals (*p* < 0.001) (Table 2).

No participants demonstrated active hepatitis B virus infection during enrollment.

### Assessment of hepatic fibrosis by transient elastography

Transient elastography demonstrated increased liver stiffness in 75% of ALL survivors, predominantly corresponding to moderate fibrosis (F2). ALL survivors demonstrated significantly higher fibrosis stages compared with controls (Table 3). Mean liver stiffness measurements were also significantly higher in survivors than controls (6.9 ± 2.3 vs. 3.2 ± 0.9 kPa, *p* < 0.001).

**Table 3:** Distribution of the fibrosis stages among ALL survivors and controls according to Fibroscan

Fibrosis stage, *n* (%)	ALL survivors (*n* = 159)	Controls (*n* = 99)	Test of significance	*P*
F0	40 (25)	99 (99)	*χ*^2^ = 134.6	< 0.0001
F1	32 (20)	0	*χ*^2^ = 19.9	< 0.0001
F2	74 (47)	0	*χ*^2^ = 65.8	< 0.0001
F3	3 (2)	0	Fisher’s exact test	0.08
F4	10 (6)	0	*χ*^2^ = 10.6	< 0.0001
LSM (Kpa), mean ± SD	6.9 ± 2.3	3.2 ± 0.9	*t* = − 11.1	< 0.0001

*χ*^*2*^, chi-square test for categorical variables. Fisher’s exact test was used when expected cell frequencies were < 5

*t*, Student’s *t*-test for comparison of means

### Assessment of hepatic fibrosis by ELF score

**Fig. 1:**
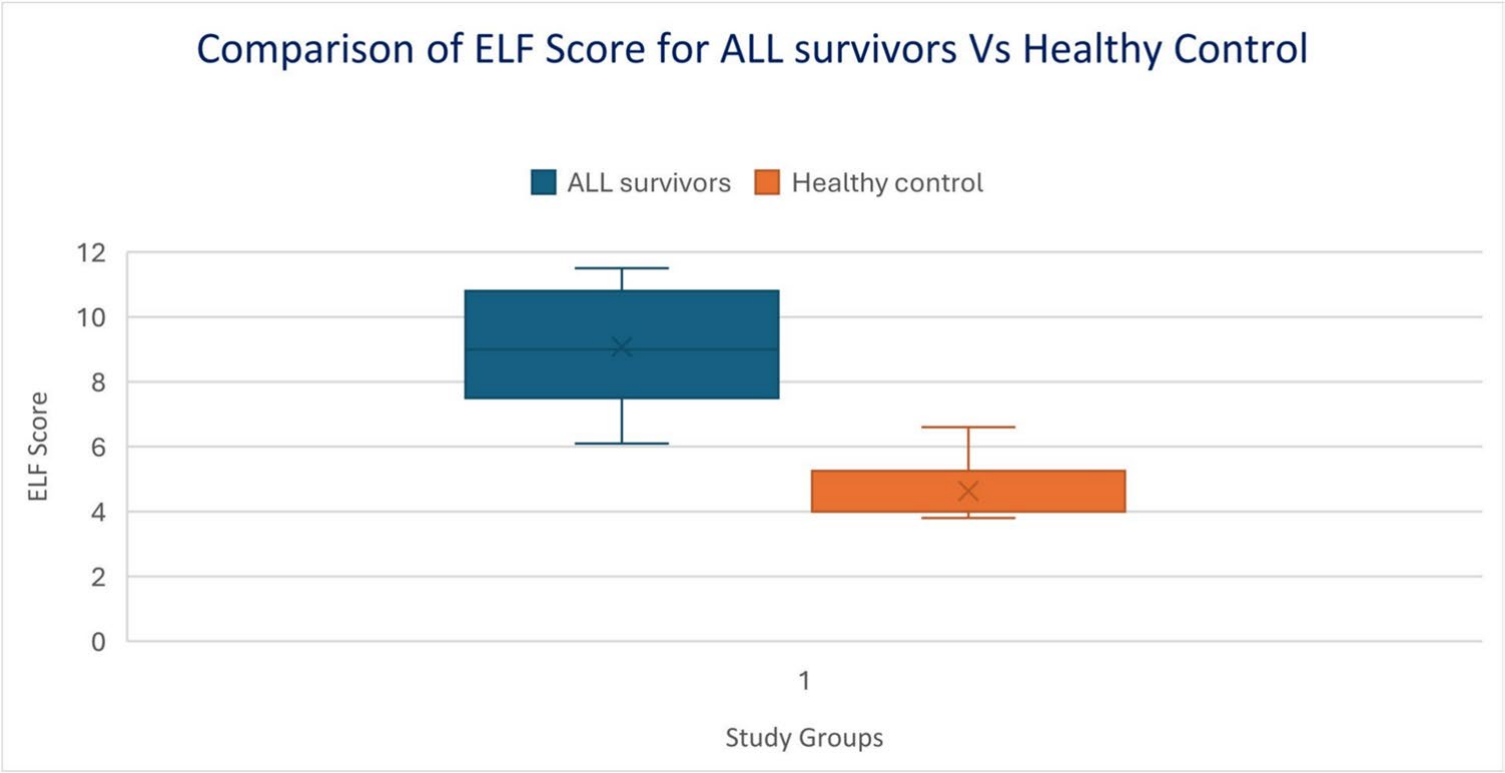
Comparison of ELF score for ALL survivors vs. healthy control

The ELF score was significantly higher in ALL survivors than in controls (9.3 ± 1.2 vs. 4.5 ± 1.03, *p* < 0.001) (Fig. 1).

Based on ELF classification, survivors had a significantly higher prevalence of moderate, severe, and advanced fibrosis compared with none in controls (Table 4).

**Table 4:** Distribution of the fibrosis stages among ALL survivors and controls according to ELF score

Fibrosis stage, *n* (%)	ALL survivors (*n* = 159)	Controls (*n* = 99)	Test of significance	*P*
F0–F1	81(51)	99(100)	*χ*^2^ = 70	< 0.0001
F2	68(43)	0	*χ*^2^ = 58.6	< 0.0001
F3–F4	10 (6)	0	*χ*^2^ = 6.2	0.021
ELF score, mean ± SD	9.3 ± 1.2	4.5 ± 1.03	*t* = − 32.94	< 0.0001

*χ*^*2*^, chi-square test for categorical variables; *t*, Student’s *t*-test for comparison of means

**Fig. 2:**
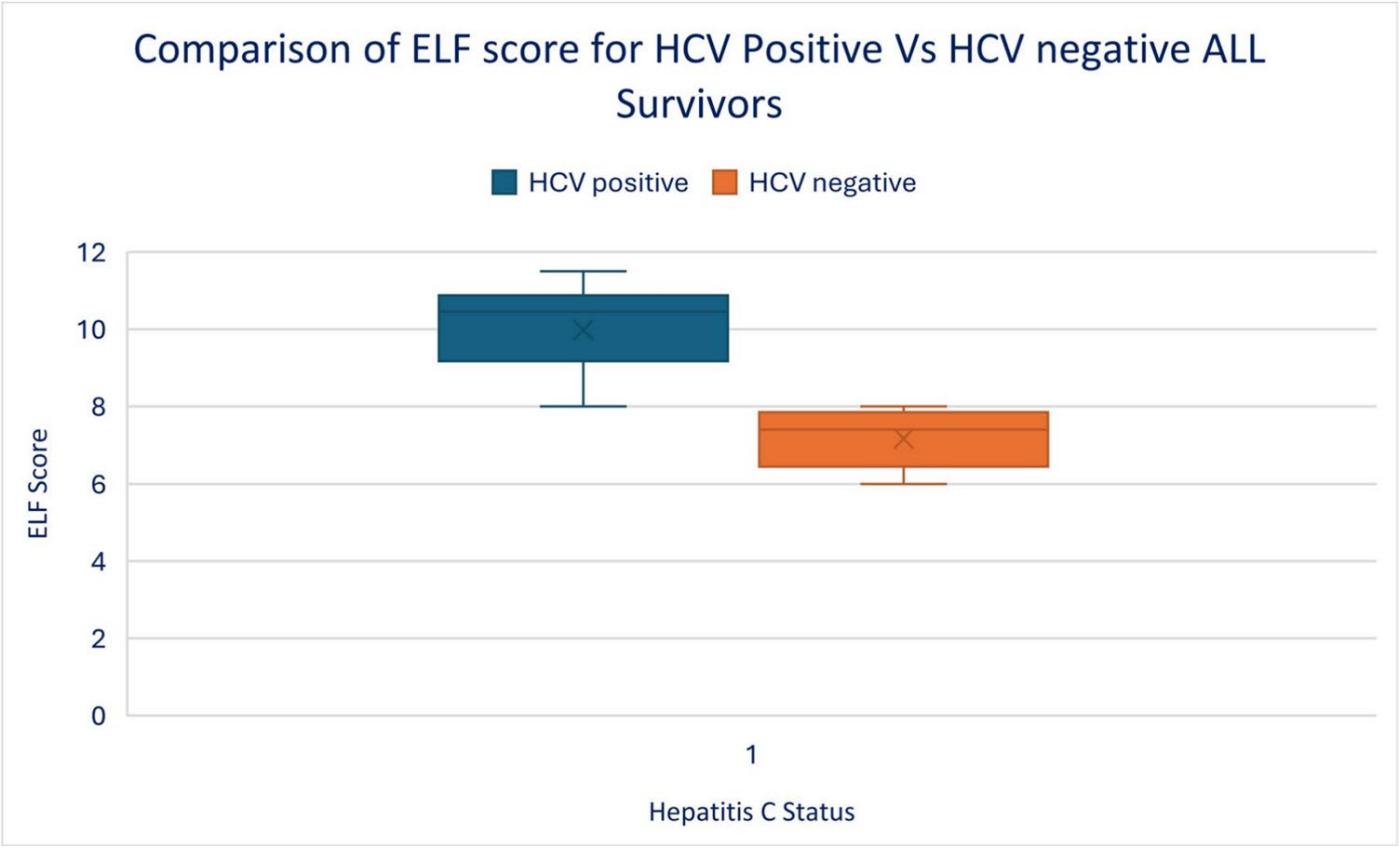
Comparison of ELF score for HCV-positive vs. HCV-negative ALL survivors

Mean ELF score was significantly higher among HCV-positive or previously treated survivors compared with HCV-negative survivors (10.5 ± 1.5 vs. 7.8 ± 1.2, *p* < 0.001) (Fig. 2), further supporting the association between prior HCV exposure and persistent hepatic fibrosis.

### Combined TE/ELF assessment

Combined TE/ELF assessment classified 45% of survivors as having mild fibrosis, 49% as moderate, severe fibrosis, and 6% as advanced fibrosis. Agreement between TE and ELF was fair (Cohen’s *κ* = 0.34–0.39), with a significant positive correlation between both methods (Table 5).

**Table 5:** Correlation between fibrosis stage and the studied variables among the survivors

Variable	Grade of fibrosis	
	rho	*P*
Age at enrollment	0.286	0.01*
Age at diagnosis	0.02	0.88
Weight	0.226	0.044
Height	0.209	0.062
BMI	0.129	0.25
Follow-up	0.105	0.35
HB	− 0.168	0.13
WBCs	0.384	< 0.001*
PLTs	− 0.113	0.32
ALT	0.042	0.71
AST	0.113	0.32
Creatinine	0.063	0.57
Urea	0.327	0.003*
ELF score	0.353	= 0.001*

### Correlation analysis

Fibrosis stage showed significant correlations with age at enrollment, weight, white blood cell count, urea level, and ELF score (Table 5).

### Association between HCV and fibrosis

HCV exposure was strongly associated with fibrosis severity. All cases with severe fibrosis were either currently HCV-positive (80%) or previously infected and treated (20%). No cases of severe fibrosis were observed among HCV-negative participants.

Among patients with moderate fibrosis, 99% had a history of HCV infection, while more than half of those with no or mild fibrosis had never been infected with HCV (Table 6).

**Table 6:** Relation between HCV status and stage of fibrosis among ALL cases

HCV status, *n* (%)	Severe/advanced F3–F4	Moderate F2	No/mild fibrosis F0–F1	Total
Currently positive	8 (80)	0	0	8 (5)
Previously HCV-positive, currently PCR-negative	2 (20)	76 (99)	33 (46)	111 (70)
Was negative	0 (0)	1 (1)	39 (54)	40 (25)
Odd ratio (*P*)	24.7 (0.02)	89.8 (< 0.000)		
Total	10 (6)	77 (49)	72 (45%)	159 (100)

ELF scores were significantly higher among survivors with current or previous HCV infection compared with HCV-negative survivors (Fig. 2).

## Discussion

The present study evaluated hepatic fibrosis among pediatric survivors of acute lymphoblastic leukemia (ALL) using non-invasive methods, namely transient elastography (TE) and enhanced liver fibrosis (ELF) score. Our findings demonstrate a significantly higher prevalence of hepatic fibrosis among ALL survivors compared with healthy controls, supporting the hypothesis that this population is at increased risk of long-term hepatic complications.

The high prevalence of fibrosis observed in our cohort (75% by TE) appeared to be strongly associated with previous HCV exposure rather than chemotherapy alone. Nearly all patients with moderate or severe fibrosis had current or previous HCV infection, whereas only one HCV-negative survivor demonstrated moderate fibrosis. These findings suggest that persistent hepatic fibrosis in this cohort was predominantly related to HCV-associated liver injury, consistent with previous studies in childhood cancer survivors with HCV infection [5, 31].

A notable finding of this study is the strong association between HCV exposure and fibrosis severity. All cases with severe fibrosis were either currently HCV-positive or had a history of treated HCV infection, while no severe fibrosis was observed among HCV-negative individuals. Similarly, nearly all patients with moderate fibrosis had prior HCV exposure. These findings further emphasize the critical role of HCV as a major risk factor for hepatic fibrosis in this population, in agreement with previous studies [5, 31].

Although treatment with direct-acting antivirals (DAAs) resulted in a high rate of viral clearance (93%), a substantial proportion of patients continued to exhibit varying degrees of fibrosis. This may reflect pre-existing liver damage prior to antiviral therapy, as baseline fibrosis was not assessed in most cases. Previous studies have reported variable outcomes regarding fibrosis regression following DAA therapy in pediatric populations [32, 33].

The significantly higher ELF scores observed among HCV-positive or previously treated patients further support the association between viral exposure and fibrotic progression. This finding further supports the predominant contribution of HCV exposure to persistent hepatic fibrosis in this cohort.

While HCV-related liver disease generally progresses more slowly in children than in adults, underlying immunosuppression and prior cancer therapy may contribute to accelerated disease progression in ALL survivors [34, 35].

The use of combined non-invasive methods in this study represents a key strength. TE provides a rapid and reliable assessment of liver stiffness, while the ELF score reflects extracellular matrix turnover. Their combined use improves diagnostic accuracy and helps overcome the limitations associated with each modality when used alone [17, 24]. The fair agreement observed between TE and ELF in our cohort is consistent with previous studies evaluating combined non-invasive approaches [17].

The presence of hepatomegaly and splenomegaly among ALL survivors in our study may be attributed to chronic HCV infection and its sequelae, including portal hypertension and hepatic fibrosis. Elevated liver enzymes (ALT and AST) further support the presence of ongoing hepatic injury in this population [28, 35].

This study has several limitations. First, its cross-sectional design precludes assessment of temporal changes in fibrosis and limits causal inference. Second, the absence of baseline fibrosis data before DAA therapy restricts the evaluation of treatment-related fibrosis regression. Third, liver biopsy, the reference standard, was not performed; however, this was intentional given the study’s focus on non-invasive assessment. In addition, variability in the interval between ALL diagnosis, completion of therapy, and fibrosis assessment may have influenced the observed fibrosis severity.

Despite these limitations, this study provides valuable insights into hepatic fibrosis in pediatric ALL survivors and highlights the clinical utility of combining TE and ELF as a non-invasive, practical approach for fibrosis assessment and follow-up.

Future longitudinal studies evaluating fibrosis status at ALL diagnosis, completion of chemotherapy, HCV detection, and long-term follow-up are warranted to better define the temporal progression of hepatic fibrosis in this population.

## Conclusions

Persistent hepatic fibrosis in pediatric survivors of acute lymphoblastic leukemia appears to be strongly associated with current or previous hepatitis C virus exposure. Despite successful antiviral treatment, residual liver fibrosis remains a significant concern in this population.

The combined use of transient elastography and enhanced liver fibrosis score may provide a reliable, non-invasive approach for early detection and monitoring of hepatic fibrosis, potentially reducing the need for liver biopsy.

Long-term follow-up and early screening strategies are recommended to prevent progression to advanced liver disease and its associated complications.

## Data Availability

All data generated or analyzed during this study are included in this published article.

## Abbreviations

ALL: Acute lymphoblastic leukemia
TE: Transient elastography
ELF: Enhanced liver fibrosis
HCV: Hepatitis C virus
HA: Hyaluronic acid
PIIINP: Amino-terminal propeptide of type III collagen
TIMP-1: Tissue inhibitor of metalloproteinase-1
IRB: Institutional Review Board
ALT: Alanine aminotransferase
AST: Aspartate aminotransferase
LSM: Liver stiffness measurement
PCR: Polymerase chain reaction
WBC: White blood cells
SPSS: Statistical Package for the Social Sciences
DAA: Direct-acting antiviral

## References

[1] El-Baz HA, Tamer EM, Elabd EM, Ramadan A, Elharoun AS, Elmorsy EA et al (2013) Serum adiponectin and resistin levels in de novo and relapsed acute lymphoblastic leukemia children patients. Iran J Public Health 42(5):504–509 23802108 PMC3684459

[2] Rabin KR, Poplack DG (2011) Management strategies in acute lymphoblastic leukemia. Oncology (Williston Park) 25(4):328–335 21618954

[3] Oeffinger KC, Hudson MM, Landier W (2009) Survivorship: childhood cancer survivors. Prim Care 36(4):743–780 19913185 10.1016/j.pop.2009.07.007

[4] Shepard CW, Finelli L, Alter MJ (2005) Global epidemiology of hepatitis C virus infection. Lancet Infect Dis 5(9):558–567 16122679 10.1016/S1473-3099(05)70216-4

[5] Stallings-Smith S, Krull KR, Brinkman TM, Hudson MM, Ojha RP (2015) Long-term follow-up for cirrhosis among pediatric cancer survivors with hepatitis C virus infection. J Clin Virol 71:18–21 26370309 10.1016/j.jcv.2015.07.306 PMC4570969

[6] Bedossa P, Dargère D, Paradis V (2003) Sampling variability of liver fibrosis. Hepatology 38(6):1449–1457 14647056 10.1016/j.hep.2003.09.022

[7] Bedossa P (1994) Intraobserver and interobserver variations in liver biopsy. Hepatology 20(1):15–20 8020885

[8] Rockey DC, Caldwell SH, Goodman ZD, Nelson RC, Smith AD (2009) Liver biopsy. Hepatology 49(3):1017–1044 19243014 10.1002/hep.22742

[9] Cholongitas E, Senzolo M, Standish R, Marelli L, Quaglia A, Patch D et al (2006) Systematic review of liver biopsy quality. Am J Clin Pathol 125(5):710–721 16707372 10.1309/W3XC-NT4H-KFBN-2G0B

[10] Bedossa P, Carrat F (2009) Liver biopsy: the best, not the gold standard. J Hepatol 50(1):1–3 19017551 10.1016/j.jhep.2008.10.014

[11] Cossiga V, La Civita E, Bruzzese D, Guarino M, Fiorentino A, Sorrentino R et al (2022) Enhanced liver fibrosis score as a biomarker in HCV. Front Pharmacol 13:891398 36059971 10.3389/fphar.2022.891398 PMC9428144

[12] Papastergiou V, Tsochatzis E, Burroughs AK (2012) Non-invasive assessment of liver fibrosis. Ann Gastroenterol 25(3):218–225 24714123 PMC3959378

[13] Lichtinghagen R, Pietsch D, Bantel H, Manns MP, Brand K, Bahr MJ (2013) The ELF score: normal values and cut-offs. J Hepatol 59(2):236–242 23523583 10.1016/j.jhep.2013.03.016

[14] Parkes J, Guha IN, Roderick P, Harris S, Cross R, Manos MM et al (2011) Performance of serum marker panels for liver fibrosis in chronic hepatitis C. J Viral Hepat 18(1):23–31 20196799 10.1111/j.1365-2893.2009.01263.x

[15] Fernandez M, Trépo E, Degré D, Gustot T, Verset L, Demetter P et al (2015) Fibroscan diagnostic performance in chronic liver disease. Eur J Gastroenterol Hepatol 27(9):1074–1079 26011235 10.1097/MEG.0000000000000392

[16] Wong GL, Chan HL, Choi PC, Chan AW, Yu Z, Lai JW et al (2014) Non-invasive algorithm of ELF and transient elastography. Aliment Pharmacol Ther 39(2):197–206 24261924 10.1111/apt.12559

[17] Arena U, Vizzutti F, Abraldes JG, Corti G, Stasi C, Moscarella S et al (2008) Reliability of transient elastography. Gut 57(9):1288–1293 18448567 10.1136/gut.2008.149708

[18] Marcellin P, Ziol M, Bedossa P, Douvin C, Poupon R, de Lédinghen V et al (2009) Non-invasive assessment of liver fibrosis by stiffness measurement. Liver Int 29(2):242–247 18637064 10.1111/j.1478-3231.2008.01802.x

[19] Rigamonti C, Donato MF, Fraquelli M, Agnelli F, Ronchi G, Casazza G et al (2008) Transient elastography for fibrosis progression. Gut 57(6):821–827 18218676 10.1136/gut.2007.135046

[20] Castera L, Forns X, Alberti A (2008) Non-invasive evaluation of liver fibrosis using elastography. J Hepatol 48(5):835–847 18334275 10.1016/j.jhep.2008.02.008

[21] Coco B, Oliveri F, Maina AM, Ciccorossi P, Sacco R, Colombatto P et al (2007) Transient elastography influenced by ALT levels. J Viral Hepat 14(5):360–369 17439526 10.1111/j.1365-2893.2006.00811.x

[22] Sagir A, Erhardt A, Schmitt M, Häussinger D (2008) Transient elastography limitations in clinical practice. Hepatology 47(2):592–595 18098325 10.1002/hep.22056

[23] Wong GL, Chan HL, Choi PC, Chan AW, Lo AO, Chim AM et al (2013) Anthropometric factors affecting liver stiffness. Clin Gastroenterol Hepatol 11(3):295–302 23022698 10.1016/j.cgh.2012.09.025

[24] Boursier J, Vergniol J, Sawadogo A, Dakka T, Michalak S, Gallois Y et al (2009) Combination of blood tests and Fibroscan for fibrosis. Liver Int 29(10):1507–1515 19725892 10.1111/j.1478-3231.2009.02101.x

[25] Pui CH, Pei D, Campana D, Cheng C, Sandlund JT, Bowman WP et al (2011) Improved prognosis for older adolescents with acute lymphoblastic leukemia treated with the total XV protocol. J Clin Oncol 29(4):386–391 21172890 10.1200/JCO.2010.32.0325 PMC3058285

[26] Kuczmarski RJ, Ogden CL, Grummer-Strawn LM, Flegal KM, Guo SS, Wei R et al (2000) CDC growth charts: United States. National Center for Health Statistics, Hyattsville (MD) 12043359

[27] Etim NN, Williams ME, Akpabio U, Offiong EE (2014) Hematological parameters and their relevance. Agric Sci 2(1):37–47

[28] Kang KS (2013) Abnormal liver function tests in children. Pediatr Gastroenterol Hepatol Nutr 16(4):225–232 24511518 10.5223/pghn.2013.16.4.225 PMC3915727

[29] de Lédinghen V, Vergniol J (2008) Transient elastography (FibroScan). Gastroenterol Clin Biol 32(Suppl 1):58–67 18973847 10.1016/S0399-8320(08)73994-0

[30] Catanzaro R, Milazzo M, Arona S, Sapienza C, Vasta D, Arcoria D et al (2013) Diagnostic accuracy of enhanced liver fibrosis score. HPB (Oxford) 15(5):500–507 10.1016/s1499-3872(13)60079-x 24103280

[31] El-Raziky MS, Halawa EF, Draz IH, Ali MS (2015) Hepatitis C in childhood cancer survivors. Pediatr Hematol Oncol 32(2):138–145 25264733 10.3109/08880018.2014.958885

[32] Fahmy DM, Shokeir M, El Zeiny SM, Jonas MM, Abdallah A (2021) Liver stiffness after direct-acting antivirals in children with chronic hepatitis C. J Pediatr 231:110–116 33347957 10.1016/j.jpeds.2020.12.031

[33] Mogahed EA, El-Karaksy H, Abdullatif H, Yasin NA, Nagy A, Alem SA et al (2021) Improvement of liver fibrosis in children with chronic hepatitis C after direct-acting antiviral therapy. J Pediatr 233:126–131 33577805 10.1016/j.jpeds.2021.02.012

[34] Leung DH, Squires JE, Jhaveri R, Kerkar N, Lin CH, Mohan P et al (2020) Hepatitis C in children: current perspectives. J Pediatr Gastroenterol Nutr 71(3):407–417 32826718 10.1097/MPG.0000000000002814

[35] Begum F, Mazumder MW, Nahid KL, Jesmin T, Musabbir N (2023) Hepatitis C virus infection in children. Egypt Pediatr Assoc Gaz 71(1):17

